# eDNA-Based Early Detection Illustrates Rapid Spread of the Non-Native Golden Mussel Introduced into Beijing via Water Diversion

**DOI:** 10.3390/ani14030399

**Published:** 2024-01-27

**Authors:** Wei Guo, Shiguo Li, Aibin Zhan

**Affiliations:** 1 Beijing Hydrology Center, Beijing 100089, China; guowei9237@hotmail.com; 2 Research Center for Eco-Environmental Sciences, Chinese Academy of Sciences, Beijing 100085, China; sgli@rcees.ac.cn; 3 University of Chinese Academy of Sciences, Chinese Academy of Sciences, Beijing 100049, China

**Keywords:** early detection, environmental DNA, golden mussel *Limnoperna fortunei*, invasive mollusk, water diversion

## Abstract

**Simple Summary:**

The world’s largest water diversion, the South-to-North Water Transfer Project (SNWTP) in China, diverts water from the Danjiangkou Reservoir in the Yangtze River basin to major cities such as Beijing and Tianjin in North China. This water diversion has effectively created an “invasion highway”, facilitating the spread of non-native invasive species such as golden mussels (*Limnoperna fortunei*) to aquatic ecosystems in Beijing. Despite the significance of this development, the dynamics of the spread and colonization patterns of golden mussels in Beijing have remained largely unexplored. In this study, we used environmental DNA (eDNA)-based early detection to conduct comprehensive surveillance across all water bodies in five river basins from 2020 to 2023. Our results revealed a rapid spread of golden mussels in Beijing over the past four years. Starting with four infested sites identified through field surveys in 2019, the count of sites exhibiting positive eDNA signals experienced a gradual rise. Between 2020 and 2022, an additional 10–13 sites were confirmed, followed by a substantial increase to 28 additional sites in 2023. Conventional field surveys confirmed the presence of golden mussels at 16 sites between 2020–2023. To date, golden mussels have rapidly expanded their presence to four out of the five investigated river basins, with only the Daqing River remaining uninfested.

**Abstract:**

The world’s largest water diversion, the South-to-North Water Transfer Project (SNWTP) in China, has created an “invasion highway” to introduce invasive golden mussels (*Limnoperna fortunei*) from the Yangtze River basin to Beijing. To examine the spread and colonization patterns of this newly introduced invasive species, we conducted comprehensive environmental DNA (eDNA)-based early detection and conventional field surveys across all water bodies in five river basins in Beijing from 2020 to 2023. Our results indicated a rapid spread over the past four years. Among the 130 tested sites, the number of sites with positive signals from eDNA analysis exhibited an annual increase: Commencing with four infested sites identified through field surveys in 2019, eDNA analysis detected an additional 13, 11, and 10 positive sites in 2020, 2021, and 2022, respectively, and a substantial rise comprising an additional 28 sites in 2023. Conventional field surveys detected mussels 1–3 years later than eDNA-based analysis at 16 sites. Across all 16 sites, we detected a low population density ranging from 1 to 30 individuals/m^2^. These findings collectively indicate that the invasions by golden mussels in Beijing are still in their early stages. To date, golden mussels have successfully colonized four out of the five investigated river basins, including the Jiyun River (22.2% positive sites), North Canal River (59.6% positive sites), Chaobai River (40% positive sites), and Yongding River (63.6% positive sites), with the North Canal River and Yongding River being the most heavily infested. Currently, only the Daqing River basin remains uninfested. Given the significant number of infested sites and the ongoing transport of large new propagules via SNWTP, further rapid spread and colonization are anticipated across aquatic ecosystems in Beijing and beyond. Consequently, we call for the proper implementation of effective management strategies, encompassing early detection, risk assessment, and the use of appropriate control measures to mitigate the potential ecological and economic damages in invaded ecosystems.

## 1. Introduction

Owing to a combination of natural and anthropogenic activity-derived stressors, water scarcity has evolved into a significant global challenge, impacting a growing number of regions throughout all continents [1]. By 2025, an estimated 1.8 billion people are anticipated to reside in countries or regions experiencing absolute water scarcity (see United Nations water scarcity at http://www.un.org/waterforlifedecade/scarcity.shtml, accessed on 30 December 2023). The water scarcity crisis is particularly pronounced in densely populated areas, especially mega cities situated in arid or semiarid regions [2]. In order to alleviate water scarcity, water diversion projects have been initiated since the early 19th century, aiming to transfer water from regions of relative abundance to those facing scarcity [3,4].

**Figure 1:**
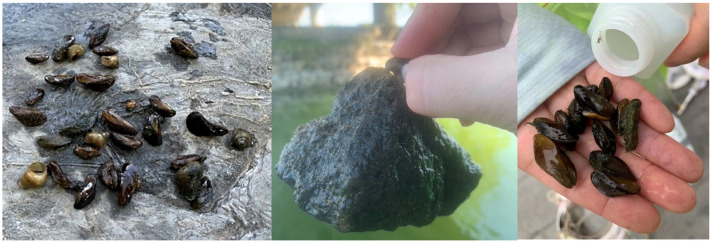
The adults of the invasive golden mussel *Limnoperna fortunei* collected across water bodies in Beijing.

Multiple mega cities in North China, such as Beijing and Tianjin, are contending with water scarcity due to rapid population growth, urbanization, and climate change [5]. Illustratively, the total per capita water availability in Beijing stands at merely 170 m^3^ before water diversions, falling significantly below the United Nations’ absolute water scarcity threshold [5]. To foster the sustainable development of these mega cities, the South-to-North Water Transfer Project (SNWTP), the world’s largest water diversion initiative, was initiated on 12 December 2014. The water diversion project has played a crucial role in enhancing the availability and equitable distribution of water resources in North China [6]. As a result, it has provided essential support for rapid and sustainable social and economic development, with a particular focus on the Beijing-Tianjin-Hebei Metropolitan region [6]. Nevertheless, shortly after the commencement of the water diversion project, Zhan and colleagues [5] emphasized that the opening of this water diversion project could construct an “invasion highway”, facilitating the spread of non-native invasive species from the Yangtze River basin and potentially leading to detrimental effects on the recipient aquatic ecosystems in Beijing. Soon after the water diversions; studies have now confirmed that what was anticipated indeed transpired [7]. Non-native species such as fishes, mollusks, aquatic plants, and particularly the highly invasive golden mussel *Limnoperna fortune* (Figure 1) have been transported through the water diversion project and started to colonize water bodies in Beijing [7,8,9,10].

The golden mussel, presumably considered native to the Pearl River in China, has undergone widespread colonization both domestically and internationally [11]. Due to the lack of trackable records, the invasion pathway and history remain largely unclear in many regions. In China, the golden mussel has expanded its presence to Hong Kong and Taiwan and northward into the Yangtze, Huaihe, Yellow, and Haihe River basins [12,13]. This mussel species has been introduced into several Southeast Asian countries, including Cambodia, Vietnam, Laos, and Thailand, likely before the 20th century [11]. Additionally, it has been introduced to East Asian countries such as South Korea and Japan through shipping and transfers of aquaculture facilities and cultured species [14,15]. In South America, the species was introduced around 1990 through ballast water discharged into the Río de la Plata estuary by transoceanic vessels [16]. Following the initial introduction, the mussel rapidly spread to and successfully colonized in Uruguay, Paraguay, Brazil, and Bolivia [11]. In invaded habitats, this mussel species has caused large-scale biofouling issues on both natural and artificial hard substrates such as rocks and cement structures, reaching extremely high population densities [17,18]. The severe biofouling problems have resulted in significantly negative ecological and economic effects, such as the suppression of local benthic communities and the obstruction of pipes in water and power plants [17,18].

Given the extensive invasion history and economic, ecological, and environmental harms associated with golden mussels, there is a crucial need to develop prevention policies and establish effective management strategies in potentially infested areas. Early detection is especially critical for management of newly introduced non-native species, as it enables a prompt response for possible eradication and control [19,20]. The detection of invasions at an earlier stage can result in more favorable outcomes and reduced economic costs because smaller invasive populations are generally easier and less expensive to manage [21,22]. Technically, the level of rarity of new invaders and success of early detection are predominantly influenced by the strategies employed and their associated sensitivity [23,24,25].

The presence of environmental DNA (eDNA) in environments provides a cost-effective method for early detection of invasive species [23]. By analyzing the eDNA released into the surrounding environment rather than isolating the target organisms themselves, researchers can detect invasive species out of complex biological communities such as those in aquatic ecosystems [23,26]. Furthermore, the combined application of DNA barcoding and eDNA collected from environments has proven to be remarkably effective in detecting the target invasive species at extremely low abundance. For example, a meticulously designed eDNA-based PCR assay can detect the target eDNA at concentrations lower than 10^−6^ ng/μL [23,27]. Thus, eDNA-based methods have been demonstrated to be more effective and sensitive tools for early detection of invasive species when compared to conventional methods such as net tow and field surveys in aquatic ecosystems [26,27,28,29]. Owing to these technical advantages, particularly its high sensitivity, eDNA-based early detection has gained popularity in identifying invasive species at an early stage.

The implementation of SNWTP has established an unprecedented invasion highway for the introduction of golden mussels into waterbodies in Beijing. Despite this development, the dynamics of their spread and the resulting ecological impacts remain largely unexplored in newly colonized waterbodies in Beijing. In this study, we employed eDNA-based early detection to pinpoint the presence of newly introduced golden mussels in various water bodies across Beijing. Meanwhile, conventional field surveys were concurrently conducted to validate positive eDNA-based detections. Our objectives are to confirm the successful colonization of golden mussels, scrutinize the geographical extent of their spread post settlement, and demarcate infested areas to facilitate the formulation of effective management strategies.

## 2. Materials and Methods

Since 2014, our research group has initiated a comprehensive routine surveillance program encompassing all major water bodies in Beijing, including streams, rivers, and reservoirs/ponds. This program entails a thorough assessment of both water quality and biodiversity based on conventional methods [7]. In our biodiversity assessments, the absence of golden mussels was recorded from 2014–2018. However, routine field surveys detected the established populations of golden mussels at four distinct sites in 2019 [7]. Considering that colonizing species typically experience a lag time before reaching a detectable population density, it remains plausible that successful colonization by golden mussels may have occurred earlier than our initial detection in 2019, very likely soon after the operation of SNWTP. To enhance our ability to detect golden mussels at an early invasion stage, we developed eDNA-based methods [29] to conduct comprehensive surveys across all water bodies beginning in 2020.

### 2.1. eDNA Sampling

**Figure 2:**
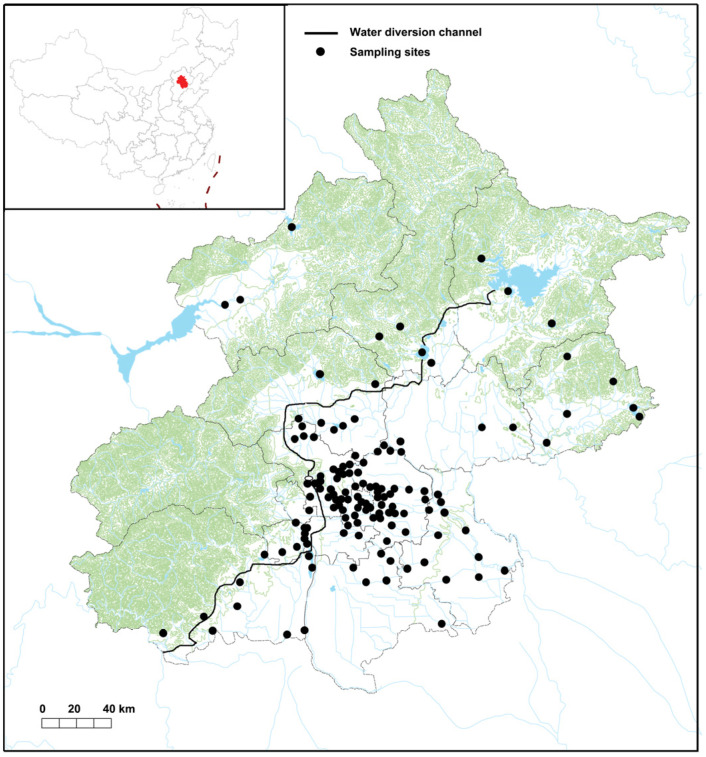
The locations of 130 sampling sites for early detection of the invasive golden mussel *Limnoperna fortunei* in Beijing from 2019 to 2023.

As golden mussels live in shallow waters with hard substrates, particularly human-made structures, we sampled eDNA from these areas in each water body from 2020 to 2023 in three seasons: spring, summer, and autumn (Figure 2). At each sampling site, a 1 L water sample was collected using a sterile bottle. The collected replicates (3 × 1 L) from each site were then amalgamated into a single 3 L sample. Subsequently, all collected samples were promptly stored at 4° C and expedited to the laboratory. Within 24 h, all water samples were subjected to filtration on 0.45 μm pore-size mixed cellulose esters (MCE) membranes (Millipore, Cambridge, MA, USA) using a vacuum pump to collect eDNA [30,31]. As a procedural negative control, 3 L of sterile pure water (Milli-Q^®^, Bedford, MA, USA) was filtered at each sampling site. All filters containing eDNA, along with the negative controls, were frozen at −80 °C until further treatment.

### 2.2. eDNA Extraction and PCR

For each sample, the filter was shredded using the well-sterilized scissors, and eDNA was extracted from the shredded membrane using the DNeasy Blood and Tissue Kit (Qiagen) following the manufacturer’s instructions. After eDNA extraction, the concentration and quality of the extracted eDNA were assessed using both the ultraviolet spectrophotometer (NanoDrop, Thermo Scientific Inc., Wilmington, DE, USA) and 2% agarose gel electrophoresis.

Each extracted eDNA sample was amplified using the primer pair B (F: AGAACCCCAGCAGTTGACATAG; R: CCACCTAGAACTGGTAGTGAAACTAAC; amplicon size = 197 bp) derived from the cytochrome *c* oxidase I (COI) gene specifically developed for eDNA-based surveys for golden mussels [29]. PCR was conducted in a 25 μL mix containing 1 × PCR buffer, 1 μL DNA extract (~10 ng eDNA), 0.05 mM of each dNTP, 0.4 mM of each primer, and 2 U of *Taq* Polymerase (Takara Bio Inc., Otsu, Japan). Thermocycler conditions included an initial denaturation at 95 °C for 5 min, followed by 35 cycles of denaturation at 95 °C for 30 s, annealing at 55 °C for 35 s, and extension at 72 °C for 1 min, with a final extension at 72 °C for 10 min. For each sample, five PCR replicates were performed to avoid biased amplifications [32,33,34]. After PCR, we loaded 5 μL of PCR products in each well of 2% agarose gels stained with ethidium bromide and then visualized using an automatic gel image analysis system. The successful detection of golden mussels was accomplished by visualizing the PCR products on agarose gels. A total of 8–10 PCR products were randomly selected for Sanger sequencing (Sangon Biotech Co., Ltd., Shanghai, China) in each year to validate the positive detections.

### 2.3. Conventional Field Surveys

Across all sampling sites (Figure 2), we executed thorough field surveys focusing on hard substrates, including manmade structures, rocks, and woods, which are conducive to golden mussel colonization. We meticulously examined hard substrates such as stones, where applicable, by flapping to search for mussels. Upon detecting golden mussels, the number of collected individuals was tallied and converted into population density (individuals/m^2^). Given that all sites were in the early stages of invasions, and mussels were confined to limited substrates, replicates for calculating average population density were not feasible. As the population density was low (see Table 1 below), we collected all found individuals at each site and immediately preserved them in a sterile plastic bottle filled with 100% ethanol (one bottle per site).

For all sites where golden mussels were detected, they were meticulously collected and subsequently subjected to morphological identification. To ensure a doubly confirmed species identification, 2–3 individuals were randomly selected from the collected specimens for molecular identification for each site. All procedures, including DNA extraction and PCR, were performed using the same protocols for eDNA. Following PCR, the obtained products underwent Sanger sequencing (Sangon Biotech Co., Ltd., Shanghai, China) to unequivocally confirm the identity of the species.

## 3. Results

### 3.1. eDNA-Based Early Detection

The eDNA-based early detection surveys spanned 130 sites (Figure 2) across five river basins (Jiyun River, North Canal River, Daqing River, Chaobai River, and Yongding River). All negative controls yielded no PCR amplification of golden mussels, indicating no external and cross-contamination throughout the entire experimental process. Sanger sequencing of all randomly selected eDNA amplicons confirmed the accurate species identification, highlighting the high specificity of the established eDNA PCR assay.

**Figure 3:**
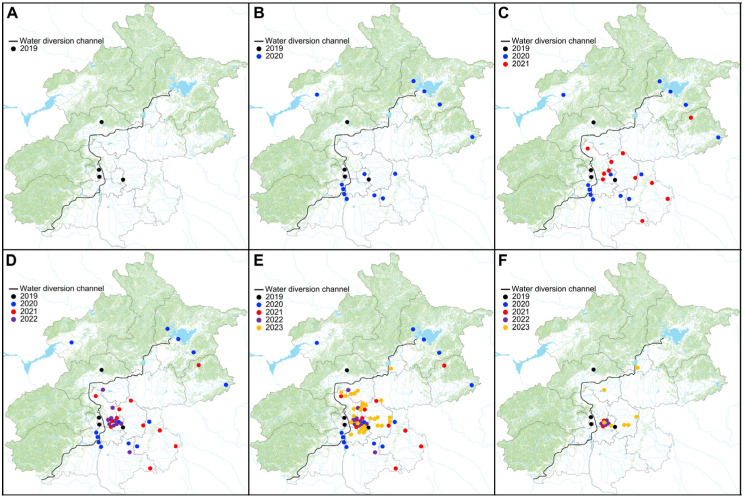
Positive signals at sites using eDNA-based early detection of the invasive golden mussel *Limnoperna fortunei* in Beijing in 2019 (**A**), 2020 (**B**), 2021 (**C**), 2022 (**D**), and 2023 (**E**). Sites where field surveys confirmed the presence of adult golden mussels are shown in different years (**F**).

Following the initial identification of colonized adult mussels by routine field surveys at four sites in the North Canal River basin in 2019 (Figure 3A), subsequent eDNA-based tests yielded positive results for these four sites, underscoring the robustness of our developed methods for early detection, as previously reported. In 2020, an additional 13 sites were confirmed as positives, and these positive sites are distributed across Jiyun River, Chaobai River, North Canal River, and Yongding River, with one, three, three, and six sites, respectively (Figure 3B). The spread trend continued in 2021 and 2022, with 11 and 10 additional positive sites observed in 2021 and 2022, respectively (Figure 3C,D); these sites are also distributed in the aforementioned river basins. In 2023, there was a notable surge in positive sites, with 28 new additions (Figure 3E). The total number of positive sites reached 66 in 2023, accounting for 50.8% (66/130) of all tested sites. This escalation in positive detections emphasizes the increasing prevalence of golden mussels across the surveyed river basins.

In terms of river basins, based on the results from eDNA analysis, by 2023, the Yongding River and North Canal River have been predominantly infested, with a total of 53 positive detections out of 89 surveyed sites (59.6%), and 7 out of 11 tested sites (63.6%), respectively. In the Chaobai River and Jiyun River basins, 4 out of 10 (40%) and 2 out of 9 sites (22.2%), respectively, returned positive results. Notably, the Daqing River basin has remained uninfested so far.

### 3.2. Conventional Field Surveys

Following Sanger sequencing, all randomly selected individuals were unequivocally identified as golden mussels, exhibiting a sequence similarity >99%. In 2020, no golden mussels were detected in any sites that had tested positive through eDNA (Table 1; Figure 3F). In 2021, golden mussels were observed at two sites: Songlin Dam in the Beihucheng River and Erre Dam in the Yongyin Channel. However, the colonizing population density was notably low, with only one individual/m^2^ recorded for both sites (Table 1; Figure 3F). In 2022, mussels were detected at nine sites (Table 1; Figure 3F). Consistent with previous years, the population density remained low, ranging from one to seven individuals/m^2^, except for one site—West Yuyuan Lake, where the population density was notably higher at 30 individuals/m^2^ (Table 1; Figure 3F). In 2023, mussels were detected at 11 sites, and as per the usual results, we detected a low population density ranging from one to seven individuals/m^2^ (Table 1; Figure 3F).

Across all sites, eDNA consistently yielded positive signals 1–3 years earlier than conventional field surveys (Table 1). For instance, at the Shahe Dam site in the Wenyu River, eDNA exhibited positive signals as early as 2021, preceding the detection of mussels through field surveys by two years (i.e., 2023; Table 1). Analogous patterns were observed at various other sites, such as Beiguan Bridge in the Xiaozhong River and Gaobeidian Dam in the Tonghui River (Table 1). For several sites such as Qijiahuozi in the Tucheng River, Longtan Dam in the Nanhucheng River, Luodao Village in the Yongyin Channel, and Erre Dam in the Yongyin Channel, the presence of mussels was confirmed by both eDNA and field surveys. However, positive signals were only indicated by eDNA in the subsequent year. For other sites such as Longbei Village in the Jingmi Channel, where positives were confirmed by both methods, no positive signals were detected by either method in the subsequent year (Table 1).

## 4. Discussion

### 4.1. Rapid Spread and Colonization of Golden Mussels in Beijing

Following the confirmation that the SNWTP serves as an invasion highway for the introduction of diverse taxa such as fishes, aquatic plants, and golden mussels [5,7,11,35], eDNA-based early detection here reveals a rapid and widespread proliferation of golden mussels across various water bodies in Beijing (Figure 3). Particularly, in 2023, we observed a substantial increase in positive sites, with the total number exceeding 50% of all tested water bodies (Figure 3). The eDNA-based detection of golden mussels was further substantiated by field surveys, providing a dual confirmation of the colonization by golden mussels at specific sites (Table 1). The rapid spread and colonization of golden mussels in 2023 can be attributed to at least two crucial factors. Firstly, the establishment of golden mussel populations in multiple water bodies across different river basins has created potential sources for further spread to nearby sites. The observed patterns align with available evidence from related studies, suggesting that newly established populations can act as sources for future invasions, rapidly pushing invasion fronts forward [36,37]. Secondly, as of 13 November 2023, SNWTP has redirected more than 60 billion m^3^ of water into North China, with an average of >6.7 billion m^3^ annually (all data from reports from the SNWTP office at http://nsbd.mwr.gov.cn, accessed on 30 December 2023). This substantial volume of water diverted from its origin (Danjiakou Reservoir in Yangtze River basin) can carry a substantial load of larvae [28,38], representing significant propagule pressure for further future colonization in both infested and uninfested water bodies within the recipient ecosystems in Beijing. Supported by theoretical, conceptual, and experimental evidence, multiple introductions with mass propagules largely ensure the invasion success [39,40,41,42]. With a significant proportion of sites testing positive (Figure 3; Table 1) and continuous transport of a huge number of new propagules, further rapid spread and colonization by this highly invasive mussel are expected across aquatic ecosystems in Beijing if effective management strategies are not properly implemented.

Among the five investigated river basins, the North Canal River and Yongding River are the most infested ones so far (Figure 3). Since the initial observation of golden mussels by our surveillance program in 2019, this highly invasive mussel has swiftly propagated across these entire river basins over the past five years. Golden mussels have spread and colonized the Jiyun River basin, with one positive site identified in 2020 and an additional site in 2021. Similarly, in the Chaobai River basin, the invasive mussels have successfully colonized, with three positive sites identified since 2020 (Figure 3). Amazingly, by 2023, 59.6% and 63.6% of the sites tested positive according to eDNA analysis in these two river basins (Figure 3). Such an observed pattern supports the common realization that once invasive species establish themselves in newly invaded habitats, there is an expectation of further rapid spread to previously uninfested areas if effective management measures are not implemented. So far, the Daqing River basin remains uninfested by golden mussels. Currently, the reasons for non-colonization in the Daqing River remain largely unclear. One plausible explanation is that Daqing River in Beijing serves as its upstream, and most of the surveillance sites are predominantly situated in mountainous areas. The limited or possibly absent introductions of propagules may constrain the success of colonization in this particular river basin in Beijing. However, recognizing the potential threat, we urgently advocate for proactive actions and the implementation of effective management programs to prevent the further spread of golden mussels into the Daqing River basin.

Following their introduction via the SNWTP, the spread of golden mussels among water bodies in Beijing exhibits diverse dispersal dynamics. The expansions to adjacent connected sites along rivers and streams in different years (Figure 3) signify natural dispersal, propelled by water currents. However, the number of connected sites is limited (Figure 2). As our routine surveillance program has strategically placed sampling sites across different types of water bodies with limited connections to obtain a comprehensive assessment of biodiversity, the rapid spread among those nonconnected water bodies illustrates the “jump” dispersal as one of the dominant dispersal dynamics. Such a dispersal mode has been observed in other habitats invaded by golden mussels, such as those in South America [40]. While human-mediated transportation of propagules, possibly facilitated by ballast water and/or ship hull fouling, may explain “jump” dispersal in South America [40], recreational boating is unlikely to be a major vector for the rapid spread of golden mussels in Beijing due to the minimal activity. Instead, “jump” dispersal can be facilitated by human activities such as the transfer of construction materials for hydraulic engineering and the transportation of plants and animals for ecological restoration. Given the widespread ecological restoration efforts in Beijing for the sustainable development of aquatic ecosystems, adults and juveniles attached to construction materials, plants, and animals can be easily transferred to uninfested areas. Additionally, as supported by related studies, other animals, such as aquatic birds, may carry mussels overland and introduce them to uninfested sites [43]. Consequently, the swift dissemination of golden mussels among water bodies in Beijing is a synergistic outcome of both natural dispersal, primarily driven by water currents and hitchhikers of other animals such as aquatic birds, and human activity-mediated dispersal, facilitated by various anthropogenic factors such as construction material transfers and ecological restoration practices.

### 4.2. Early Stages of Invasions by Golden Mussels in Beijing

Based on our comprehensive field surveys, the density of invasive populations has remained relatively low, ranging from 1 to 30 individuals/m^2^ (Table 1). When compared with established populations in other invaded habitats, where mussel beds form with densities often >200,000 individuals/m^2^ [2,44,45], the current population density in Beijing is significantly lower. Meanwhile, during our field surveys, we observed that many hard substrates, such as bare rocks and manmade cement structures, remain uncolonized at positive sites. In addition, at the site of Longbei Village in the Jingmi Channel, positive results were obtained through both eDNA analysis and field surveys in 2022; however, no positive results were detected in 2023 (Table 1). This negative outcome implies that the small invading population at this site either went extinct or reached a density below the detection threshold of eDNA analysis. These findings collectively suggest that the invasions of golden mussels in Beijing are still in their early stages. Often, invasive populations undergo a lag time throughout the invasion process, from the introduction and establishment to the final population explosion. The duration of these time lags can significantly vary among different organisms and ecosystems in various invasion stages, ranging from several years to several hundred years [46,47]. In the case of the golden mussel invasion in Beijing, the rapid spread has manifested during the very early stages of the invasion, effectively compressing or, in some instances, even negating the typical lag time observed from the introduction to subsequent spread.

Shortly after the initiation of the SNWTP, the prevailing arguments favoring a low invasion risk by golden mussels in Beijing gained traction, mainly because studies in other regions revealed that golden mussels experienced 100% mortality after 38 days at 5–7 °C, and 5 °C was therefore regarded as a critical threshold for their survival during winter, e.g., [48]. Consequently, it was inferred that golden mussels were unlikely to endure cold winter weather in North China, especially in Beijing, given that the water temperature in these regions typically falls below 5 °C. Zhan and colleagues [5,7] proposed that certain evolutionary processes, such as phenotypic plasticity and rapid environmental adaptation of golden mussels, could occur swiftly. These evolutionary processes, in turn, may contribute to their survival and subsequent spread and population expansion, leading to population exploration in North China, including Beijing. To witness the outcomes of these evolutionary processes, they designed a wintering test by caging adult golden mussels in a reservoir in Beijing [37]. The test yielded surprising results—golden mussels collected from Beijing demonstrated high resilience by surviving for 6 days at <1 °C, 41 days at <2 °C, and an impressive 108 days at <5 °C, resulting in an overall survival rate of 27% [37]. Surprisingly, during the field surveys in early spring, we discovered live adult golden mussels in shallow water bodies that had been completely frozen during the winter. These surprising findings unequivocally demonstrate that golden mussels have effectively adapted to cold winter conditions in Beijing. Successful winter survival suggests that populations that have well adapted to extreme environments can rapidly advance invasion fronts. Moreover, the adaptation to cold conditions may play a crucial role in decreasing the time lag typically associated with population explosions.

### 4.3. eDNA-Based Early Detection

The success of early detection in invasive species hinges significantly on the strategies employed and their corresponding sensitivity, and the choice of a strategy largely determines when and at which stage invaders can be effectively identified [23,49,50]. Conventional early detection strategies, which typically rely on the “catch and examine” method for target organisms, often face challenges in effectively detecting newly invading species, primarily due to sampling difficulties for those rare taxa [50,51]. In comparison to conventional field surveys, eDNA-based analysis has the capability to detect invading species at very low abundances [26,27,28,29], often enabling the identification of target organisms far before traditional strategies. The findings in this study provide direct evidence supporting this technical conclusion, demonstrating that eDNA-based analysis is capable of detecting invading populations with low population density at the very early stages of invasions (Table 1). After eDNA detection over several years, field surveys physically identified adults at sites exhibiting positive eDNA signals (Table 1). The simultaneous use of eDNA and conventional field surveys in this context offers both cross-verification of early detection results and direct evidence affirming that eDNA-based analysis can identify invading populations at the very early stages of invasions.

When using eDNA for early detection, a critical source of potential false positives arises from eDNA pollution originating from upstream sources. Indeed, the distance of eDNA transport in nature significantly varies across aquatic ecosystems, with numerous factors, such as hydrodynamics, chemical composition, and biological elements, influencing the transport distance [52]. In the diversion channels of the SNWTP, the eDNA signals became nearly undetectable at a distance of approximately 22 km from the source [28]. Since our sampling sites are often distributed at distances greater than 22 km, and there are no confirmed golden mussel sources upstream for most positive sites, the positive eDNA signals are often real rather than false positives. Furthermore, the cross-verification through field surveys has substantiated the eDNA-based results at the sites where golden mussels were identified.

## 5. Conclusions

Shortly after the introduction of golden mussels into Beijing, their rapid spread became evident across diverse water types in four out of the five river basins. In 2023, a significant increase in positive sites was observed, with over 50% of surveyed locations displaying positive results in eDNA-based early detection. Among the investigated river basins, the North Canal River and Yongding River basins have been particularly heavily infested, accounting for over 59% and 63% of positive sites, respectively. To date, only the Daqing River basin remains uninfested. Field surveys lag behind eDNA-based analysis in detecting mussels, with confirmation of adult presence commencing in 2021. By 2023, mussels were identified at more than 10 sites. The overall population density at all positive sites has remained low, ranging from 1 to 30 individuals/m^2^, indicating that the invasions in Beijing are in their early stages. Owing to the swift dissemination and ongoing introductions of substantial propagules through the SNWTP, we anticipate further spread and colonization not only within Beijing but also extending beyond its borders.

Due to the substantial negative ecological and economic repercussions associated with golden mussels, we strongly advocate for the implementation of effective management practices across all five river basins in Beijing and beyond. Recognizing that the management of invasive species is most effective and economical at early stages, we emphasize the necessity for proactive measures. In uninfested waters and areas with potential future infestations, routine early detection, particularly employing eDNA-based methods, should be routinely conducted to facilitate risk assessment and allow managers to explore viable control options [28,53]. For ecosystems already affected by golden mussels, a range of established eradication strategies is available. These strategies include biological control such as predation using fish [2], physical prevention methods such as the use of antifouling materials/coatings and ultraviolet light [2], and chemical eradication using oxidizing chemicals and magnetic nanoparticles [54,55]. The selection of these strategies should be tailored to specific water types (e.g., lotic and lentic systems) and usage scenarios (e.g., diversion channels and natural waters) to effectively control and possibly eradicate the invasive mussels.

Due to the highly invasive nature of golden mussels, any residual propagules post eradication hold the potential to seed viable populations locally and beyond, thereby posing a risk of failure despite extensive management efforts. The timely insights provided by eDNA-based analysis are crucial in addressing the effectiveness of management and eradication efforts. The utilization of eDNA-based detection is especially effective in identifying low levels of propagules post eradication in managed water bodies, where traditional surveys struggle and often fail to detect populations of such minimal size. Therefore, eDNA-based analysis provides an effective means of evaluating eradication and other management outcomes.

## Figures and Tables

**Table 1:** Positive sites that were confirmed by conventional field surveys and the density (ind./m^2^ = individuals/m^2^) of observed colonizing populations of the golden mussel, *Limnoperna fortunei*. E, positive sites detected by eDNA analysis; C, conventional field surveys confirmed the presence of invasive golden mussels; N, negative result.

Site (Coordinate)	2020	2021	2022	2023
Shahe Dam, Wenyu River (40°00′16″ N, 116°16′55″ E)	N	E	E	E, C (1 ind./m^2^)
Qijiahuozi, Tucheng River (39°58′55″ N, 116°23′23″ E)	N	N	E, C (1 ind./m^2^)	E
Beiguan Bridge, Xiaozhong River (39°56′13″ N, 116°40′10″ E)	N	N	E	E, C (6 ind./m^2^)
Gaobeidian Dam, Tonghui River (39°54′51″ N, 116°32′8″ E)	E	N	E	E, C (2 ind./m^2^)
Longtan Dam, Nanhucheng River (39°53′13″ N, 116°27′06″ E)	N	N	E, C (1 ind./m^2^)	E
Songlin Dam, Beihucheng River (39°57′21″ N, 116°23′01″ E)	N	E, C (1 ind./m^2^)	E, C (1 ind./m^2^)	E, C (4 ind./m^2^)
Luodao Village, Yongyin Channel (39°55′27″ N, 116°18′28″ E)	N	N	E, C (3 ind./m^2^)	E
Erre Dam, Yongyin Channel (39°54′18″ N, 116°21′21″ E)	N	E, C (1 ind./m^2^)	E, C (6 ind./m^2^)	E
Longbei Village, Jingmi Channel (40°01′20″ N, 116°16′27″ E)	N	N	E, C (1 ind./m^2^)	N
Bayi Lake (39°55′11″ N, 116°19′31″ E)	N	N	N	E, C (4 ind./m^2^)
West Yuyuan Lake (39°55′24″ N, 116°19′07″ E)	N	N	E, C (30 ind./m^2^)	E, C (5 ind./m^2^)
East Yuyuan Lake (39°55′25″ N, 116°19′48″ E)	N	N	E, C (7 ind./m^2^)	E, C (7 ind./m^2^)
Zizhuyuan Lake (39°56′49″ N, 116°19′30″ E)	N	N	N	E, C (4 ind./m^2^)
Beijing Zoo Ponds (39°56′50″ N, 116°20′14″ E)	N	N	E, C (2 ind./m^2^)	E, C (1 ind./m^2^)
Gaobeidian wastewater drainage (39°53′56″ N, 116°31′51″ E)	N	N	N	E, C (2 ind./m^2^)
Huairou wastewater drainage (40°17′03″ N, 116°37′10″ E)	N	N	N	E, C (1 ind./m^2^)

## Data Availability

All data needed to evaluate the conclusions in this paper are present in the text.
